# Message-Based Communication for Heterogeneous Internet of Things Systems

**DOI:** 10.3390/s20030861

**Published:** 2020-02-06

**Authors:** Bogdan Oniga, Leon Denis, Vasile Dadarlat, Adrian Munteanu

**Affiliations:** 1 Department of Computer Science, Faculty of Automation and Computer Science, Technical University of Cluj-Napoca, Memorandumului 28, 400114 Cluj-Napoca, Romania; vasile.dadarlat@cs.utcluj.ro; 2 ETRO Department, Vrije Universiteit Brussel, Pleinlaan 2, B-1050 Brussels, Belgium; ldenis@etrovub.be (L.D.); acmuntea@etrovub.be (A.M.)

**Keywords:** heterogeneous IoT systems, network management, message-based communication, power consumption

## Abstract

The Internet of Things (IoT) domain presents a wide spectrum of technologies for building IoT applications. The requirements are varying from one application to another granting uniqueness to each IoT system. Each application demands custom implementations to achieve efficient, secure and cost-effective environments. They pose a set of properties that cannot be addressed by a single-based protocol IoT network. Such properties are achievable by designing a heterogeneous IoT system, which integrates diverse IoT protocols and provides a network management solution to efficiently manage the system components. This paper proposes an IoT message-based communication model applied atop the IoT protocols in order to achieve functional scalability and network management transparency agnostic to the employed communication protocol. The paper evaluates the proposed communication model and proves its functional scalability in a heterogeneous IoT system. The experimental assessment compares the payload size of the proposed system with respect to the LwM2M standard, a protocol designed specifically for IoT applications. In addition, the paper discusses the energy consumption introduced by the proposed model as well as the options available to reduce such impact.

## 1. Introduction

Internet of Things devises and maintains the synergy between the digital and physical environment. IoT comprises a wide variety of applications and protocols that grant uniqueness to each implementation. In such complex ecosystems, the implementations are varying in accordance with multiple factors such as environment properties, in-use protocols, ecosystem efficiency and costs. Taking into consideration all these factors, building an efficient and cost-effective heterogeneous IoT system represents a big challenge.

To achieve an effective heterogeneous IoT system, besides the multi-protocol communication interoperability, the system must provide an effective network management solution responsible for configuring, monitoring and maintaining the IoT entities agnostic to the employed communication protocol. Heterogeneous IoT systems have various uplink and downlink traffic which is caused by protocol heterogeneity, device configuration, firmware upgrade, commands execution, etc. It becomes important for the implied entities to define a common set of rules and practices to achieve semantic interoperability.

This paper proposes an IoT message-based communication (IMBC) model applied atop the IoT communication protocols to achieve network management transparency despite the transmitting protocol. IMBC is implemented at the application level and comprises a dictionary of services utilised by the devices and the server to interact and to perform the desired actions. The dictionary is one of the core components that allows management procedures such as device configuration, payload identification, device control, firmware update, transmission cycle configuration and error handling. The paper also evaluates the proposed communication model and demonstrates the functional scalability of a heterogeneous IoT system applying the IMBC model. It compares the generated payload sizes of IMBC with respect to the LwM2M standard and, last, assesses the energy consumption impact introduced by the proposed techniques.

This paper is structured as follows. Section 2 presents the related work. Section 3 describes the proposed message-based communication model. Its particularities are furthered detailed in Section 4, whereas the experimental evaluation is given in Section 5. Finally, Section 6 draws the conclusions of this work.

## 2. Related Work

Building efficient and cost-effective IoT applications presents a big challenge for developers. The protocol variety and their unique properties are the key elements taken into consideration to fulfil the application needs [1,2]. For complex IoT applications, a single-based protocol network may not suit the application requirements due to protocol limitations, forcing a multi-protocol network to cover all application particularities. To effectively handle such a heterogeneous system, it becomes important to implement a network management solution designed to manage the IoT network components.

Many approaches and solutions have been introduced in the literature which address adaption layers and integration techniques to achieve protocol interoperability in heterogeneous IoT systems. Protocol interoperability has been implemented into the most popular IoT cloud-based services [3,4]. OneM2M is a standard defined to achieve a unique architecture for IoT technologies [5]. The community and organisations are continuously expanding its capabilities. For example, J. Koo et al. [6] analyse the types of device identifiers utilised by various IoT cloud-based services (e.g., Google, IBM, Cisco, Samsung, etc.) and propose an IoT device identifier translator that translates identifiers to oneM2M format to achieve abstract device identification. A heterogeneous IoT gateway is proposed in [7], which allows package conversion between different IoT protocols. Design challenges of a heterogeneous IoT system which passively detects and tracks moving elements have been studied in [8].

The previously mentioned works focus on functionality and technical interoperability of multiple protocols at the communication level. However, the major challenge of designing heterogeneous IoT systems is to provide a network management solution and semantic interoperability between the IoT entities. The latter has been addressed in [9], which proposes an expressive ontology named IoT-O, an extension of the oneM2M standard which supports semantic data interoperability for network management and automatic interpretation of data coming from different sources. Their techniques, however, depend on the underlying transport protocol, rendering it unsuitable for an agnostic system to perform similar tasks. The Open Mobile Alliance (OMA) has released the OMA Lightweight Machine to Machine (LwM2M) protocol [10], a protocol meant to extend and match the requirements of IoT applications. LwM2M is a light, compact and resource efficient protocol implemented with the Constrained Application Protocol (CoAP), which facilitates device management and service data transfer. The LwM2M is applied successfully on devices with Internet access [11,12,13] and multiple solutions have been addressed that apply LwM2M on non-Internet devices. To implement LwM2M, such devices must adopt the Internet Protocol (IP) [14,15] or implement translators for LwM2M objects [16]. Other related work includes Smart Objects which has been defined by the Internet Protocol for Smart Objects (IPSO) Alliance. They provide object models for high level interoperability between devices [17]. The object models are applicable on any RESTful protocol since the object description is mapped into the URI path and targets IP-enabled devices. Though applicable in many settings, the usage of a RESTful protocol combined with their serialisation generates payloads, which can be deemed too large for battery-powered devices.

## 3. IoT Message-Based Communication (IMBC)

This work proposes a generic IoT message-based communication model to achieve IoT network management transparency. It defines a dictionary of semantic data services utilised by the devices and the server to interact and perform the desired actions. The dictionary of services refers to a set of object models which describe environmental information (e.g., temperature, humidity, etc.) or management procedures (e.g., device configuration, firmware update, etc.). Unlike IoT-O, the proposed message communication is independent of the underlying standards and performs management procedures agnostic to the IoT transport protocol. IMBC also serves as an alternative to the LwM2M protocol in terms of service bootstrapping. It is employed at the application level, irrespective of the underlying transport protocols, which utilise the device identifier format enforced by server integration. Unlike LwM2M, IMBC does not define Client End Point Names since the device identification is enforced and performed by the server for each integrated IoT protocol. IMBC improves over LwM2M by eliminating the need of IP or LwM2M object translators for non-Internet devices. Moreover, the IMBC serialisation format permits to construct payloads of sizes lower than that of the LwM2M and IPSO models. For clarity, we have summarised the main similarities and differences of IMBC and LwM2M in Table 1. The IMBC definitions and parsers are publicly available (https://gitlab.com/openiot/imbc).

For IMBC, the semantic data services are uniquely identified by an 8-bit identifier. The services describe specific functions that are utilised by the central manager to communicate the desired commands to the devices or to process the collected information. Those functions can for example be temperature measurements, battery level reporting, firmware update, etc. Concretely, IMBC defines services responsible for device configuration, payload identification, device control, firmware update, transmission cycle configuration and error handling. Table 2 presents the service categories and their identifier range values.

The message payloads comprise at least one semantic data service representation which in turn consists of two parts, that is, the service identifier and the service message. The service identifier determines the service for which the following service message is intended. The service message contains the actual data associated with that service. The number of services comprised by a single message varies based on the limitation of the employed IoT protocols, such as the maximum payload size. In other words, IMBC allows sending data of multiple services in one single message, thus reducing the network load. A message can for example be sent by the end-node to publish both the temperature and its battery level. As a receiver, the central manager identifies the service representations based on the service identifiers, processes the service messages accordingly and extracts the corresponding data. The message format and its characteristics are described next.

### 3.1. The Message Format

A service is described as a JavaScript Object Notation (JSON) object of which the key is the service identifier byte value represented as a hexadecimal number. The JSON object definition comprises the service name, the title, the description and the service message particularities, such as the data structure, type and unit.

**Figure 1:**
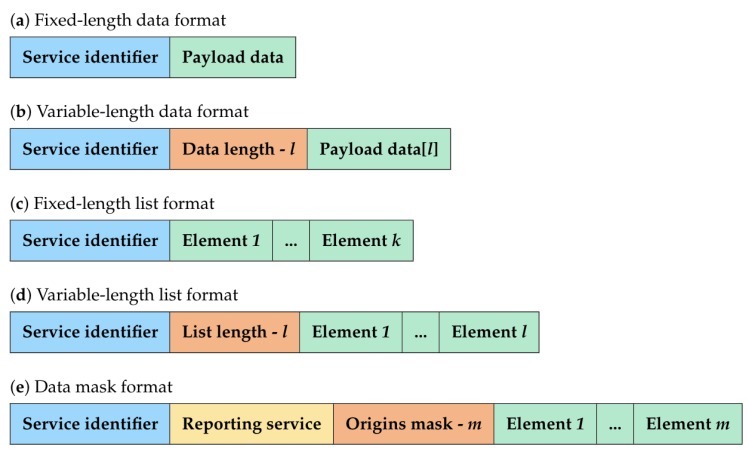
Service message formats.

As shown in Figure 1, IMBC implements five different formats for the service messages: fixed-length data, variable-length data, fixed-length list, variable-length list and data mask. We will provide more details about each hereunder.

#### 3.1.1. Fixed-Length Data Format

An object definition of a fixed length service message specifies the data length in bytes and its type. The total size of such a service message format is computed as follows,

(1) $$fld\left(s\right)=s_{id}+s_{dl},$$

with *s*, $s_{id}$ and $s_{dl}$ representing the service, service identifier length and service data length, respectively. We note that the naming of *s* and $s_{id}$ will be consistent throughout this section. Below we exemplified a temperature service object definition which defines a service message of 4 bytes length represented as floating point.


{
    "20": {
        "service": "temperature",
        "title": "Temperature",
        "description": "Temperature value in degrees Celsius",
        "type": "float",
        "length": 4,
        "unit": "°C"
    }
}
		  

**Figure 2:**
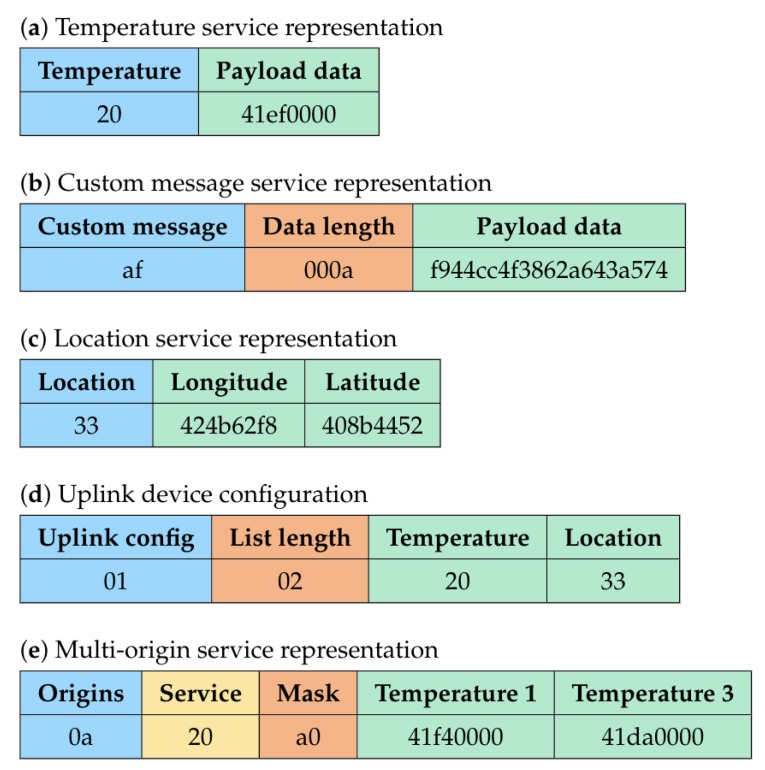
Service message formats examples.

Figure 2a shows the temperature service representation associated with the JSON object. It comprises the service identifier (“20” hexadecimal), followed by the service message (“41ef0000” hexadecimal), which in this case is a 4 byte floating point representing value “29.875”.

#### 3.1.2. Variable-Length Data Format

A service message transporting data of variable length comprises the data length, followed by the actual data. The data length is a decimal number given by the first bytes as specified by the service object definition. The service representation size is computed by

(2) $$vld\left(s\right)=s_{id}+s_{dvl}+s_{dl},$$

where $s_{dvl}$ denotes the number of bytes necessary for describing the data length. The length of the service data itself is presented as $s_{dl}$. As described below, the custom message service object defines a variable data length given by the first two bytes, resulting in a custom message of maximum 65,535 bytes long.


{
    "af": {
        "service": "custom_message",
        "title": "Custom message",
        "description": "Custom message of variable length",
        "type": "byte",
        "variable-length": 2
    }
}
		  

The corresponding data representation is given in Figure 2b. From the figure, we see that it consists of the service identifier (“af” hexadecimal) and the service message, which is split as follows, the data length (“000a” hexadecimal: 10 bytes) and the data (“f944cc4f3862a643a574” hexadecimal).

#### 3.1.3. Fixed-Length List Format

The elements of a fixed length list are extracted based on the number of elements and their size in bytes specified by the service definition. Equation (3) computes the total size of a service representation carrying data lists. It is defined as

(3) $$fll\left(s\right)=s_{id}+s_{ll}\times l_{el},$$

where $s_{ll}$ represents the service list length and $l_{el}$ symbols the data size of each element. Below an example is provided for a service which reports a location represented as a list of two float values, latitude and longitude.


{
    "33": {
        "service": "location",
        "title": "Location",
       "description": "Location value given by the latitude and longitude.",
        "list-length": 2,
        "type": "float",
        "element-length": 4,
        "unit": "°"
    }
}
		  

As shown in Figure 2c, the location service representation internally consists of two parts, the service identifier (“33” hexadecimal) followed by the service message, which is this case consists of the latitude (“424b62f8”) and the longitude (“408b4452”), having floating point values “50.84665” and “4.35209”, respectively.

#### 3.1.4. Variable-Length List Format

As will later be described in Section 3.2.1, IMBC provides three management procedures utilised for device configuration. The underlying component for these procedures is the service message format used to transport lists of variable length. Such service messages comprise the list length and the elements of the list. The payload size for such a message is given by

(4) $$vll\left(s\right)=s_{id}+s_{lvl}+s_{ll}\times l_{el},$$

where $s_{lvl}$ is the number of bytes describing the list length. $s_{ll}$ and $l_{el}$ denote the number of elements in the list and the length of each element, respectively.

The service object definition exemplified hereunder permits to transmit a list of services of maximum 256 elements, as the length is provided by the first byte which has a maximum value of 255. 


{
    "01": {
        "service": "uplink_config",
        "title": "Uplink service configuration",
        "description": "Specifies the uplink services implemented by the device",
        "variable-list-length": 1,
        "type": "byte",
        "element-length": 1
    }
}
		  

The service representation described in Figure 2d transports a list of two services. The list length is given by the first byte (“02” hexadecimal). The list itself consists of the service identifiers for temperature (“20”) and location (“33”).

#### 3.1.5. Data Mask Format

IMBC defines a data format responsible for handling data coming from multiple sources. More concretely, the data mask format carries multiple values corresponding to one specific service, which is called the reporting service. Using this format, one can send measurements of many different sensors in a single message, thus reducing the network load. The origins of the values are encoded in a binary mask. The message content of this data format contains the reporting service, the mask and the actual data. Its payload size is given by the following equation,

(5) $$dm\left(s\right)=s_{id}+s_{sr\_id}+s_{ml}+m_{b}\times \left(mf\left(s_{sr}\right)-s_{sr\_id}\right),$$

with $s_{sr}$ and $s_{sr\_id}$ representing the reporting service and reporting service identifier length. The service mask length is denoted by $s_{ml}$. The number of bits in the mask having value 1 is given by $m_{b}$. Finally, $mf(s_{sr})$ returns the data length as defined by Equations (1)–(4) of the reporting service depending on its data format.


{
    "0a": {
        "service": "origins_8",
        "title": "Service multi-origins (8-bit)",
        "description": "Maximum of 8 service reporting origins",
        "mask": 1,
        "type": "byte",
    }
}
		  

A service responsible for carrying multi-origin data is exemplified above. A message for this service is provided in Figure 2e. This particular message transmits the temperature (“20” hexadecimal) of two different sensors, of which the origin is defined by the mask (“a0” hexadecimal = “10100000” binary). The temperature values reported by the first and the third origins are “41f40000” and “41da0000”, respectively.

### 3.2. Management Procedures

IMBC defines a set of services that enable management procedures responsible for device configuration, payload identification, transmission cycle configuration, device control, firmware update, protocol set-up and error handling. This section describes the applicability of these services in the network management of heterogeneous IoT applications.

#### 3.2.1. Device Configuration

The device configuration procedure accomplishes two tasks, namely, device configuration announcement and device reconfiguration. The role of the former is to announce the implemented services at the device level whenever a device is initialised. The announcement is utilised by the server to automatically generate the necessary controls and device management functions. By implementing the device reconfiguration functions at both server and device level, the server acquires the ability to modify the device configuration at will.

IMBC defines three services for device configuration announcement: dual service configuration (“00”), uplink service configuration (“01”) and downlink service configuration (“02”). The up- and downlink services allow for up and download traffic, respectively, whereas the dual service configuration allows for both. The dual service configuration can for example be used for a thermostat where the uplink traffic is employed for reporting the temperature to the server. The downlink traffic is then utilised for adjusting the temperature threshold to indicate when the heater should start. An example where only uplink traffic is necessary can be a device only reporting its battery level. A device only needing firmware updates can serve as another example only requiring a downlink service.

#### 3.2.2. Payload Identification

This procedure identifies and processes the Payload Data Object (PDO) services that are responsible to carry the payload data. When originating from devices, the PDO’s carry reporting data such as the battery level. When sent by the server, they can carry data used for device configuration. An example can be the temperature threshold for the thermostat.

#### 3.2.3. Transmission Cycle Configuration

IMBC defines a service responsible for configuring the time period between two successive transmissions. The time period, expressed in milliseconds, is declared as an unsigned integer value which permits time period configurations with a maximum of ∼49 days.

#### 3.2.4. Device Control

IMBC also provides a service which allows the server to perform actions such as power off, restart or sleep at the device level. This service has identifier “13”. The actions are represented by a 1 byte value corresponding to the predefined actions listed in Table 3.

#### 3.2.5. Firmware Update

IMBC handles firmware updates using two services: One service targets non-Internet devices, whereas the other handles Internet-connected devices. The former has service identifier “11” and carries firmware batches of variable length. The latter uses identifier “12” and transports the firmware’s Uniform Resource Locator (URL) that points to the firmware available for download.

#### 3.2.6. Protocol Setup

The protocol set-up procedure is meant for managing the IoT protocol used for succeeding transmissions. This is mandatory for hybrid devices implementing more than one IoT protocol. The identifier for this service is “10”. The payload data comprises the desired protocol identifier chosen from the predefined list of protocols shown in Table 3. If the message sequence is “00”, the transmitting protocol is automatically chosen by the receiver.

#### 3.2.7. Error Handling

Error handling refers to the procedure of handling the error conditions present at the device level. This service is used to detect errors generated by the management procedures and maintaining device functionality. It has identifier “0a” and transports the predefined 1 byte error messages shown in Table 3.

## 4. Network Management Characteristics

IMBC achieves syntactic and semantic interoperability by implementing a common set of rules which are followed by both the server and the devices. Syntactic interoperability refers to the ability of each device within the network to communicate with one another. Note that, in this case, the term device is used broadly, and thus also includes the server. This type of interoperability is obtained by IMBC through the means of the service message formats. Semantic interoperability, on the other hand, is the result of the server and the devices implementing adaptors that automatically interpret data meaningfully by converting the service messages into system-readable representations. The syntactic and semantic interoperability allow IMBC to achieve device configuration adaptability and functional scalability.

### 4.1. Device Configuration Adaptability

Device configuration adaptability refers to the server’s ability to adapt the management functions based on the device configuration. Concretely, the server automatically generates the list of interfacing functions for a device and the available services. It implements the interfacing functions for each IMBC service, which allow the users or the integrated applications to manage the IoT devices.

**Figure 3:**
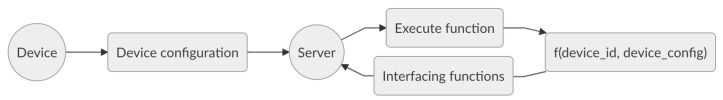
Device configuration adaptability.

As illustrated in Figure 3, the device configuration is announced whenever a device is initialised. More specifically, each device provides a list of implemented services to the server, which in turns executes a function *f* with parameters the device identifier and the device configuration. This function therefore returns a list of interfacing routines that can be used for device management. These interfacing functions play an important role in achieving functional scalability described hereunder.

### 4.2. Functional Scalability

**Figure 4:**
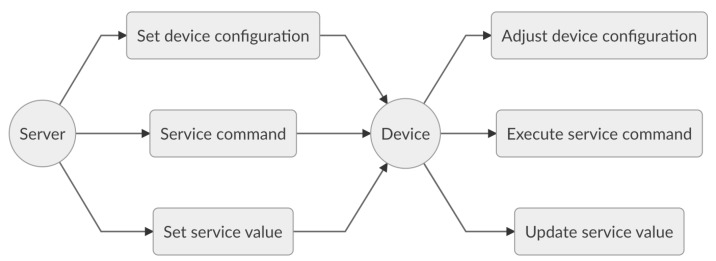
Device functional scalability.

Functional scalability refers to the ability to enhance an IoT system by extending functionalities without disrupting existing activities. Being functionally scalable is highly advisable for IoT systems, due their evolving nature and their heterogeneity. Tasks such as battery replacement for battery-powered devices, device reporting adjustments or device reachability enhancement, demonstrate the need for such scalability to manage IoT systems with minimal effort. In this context, IMBC provides services and management procedures to adjust the device configuration, to perform service commands or to update service parameters at the device level as shown in Figure 4.

Though functional scalability vastly simplifies the management of IoT systems and in addition enhances the ability to automate data representations and application interactions, it does increase the overall complexity of the application. In other words, a trade-off between the management capabilities and energy consumption is to be made. In order to reduce the overhead introduced by IMBC, we have implemented support for power profiles that can be used to reduce power consumption. We will further elaborate on this next.

### 4.3. Transmission Energy Consumption

The transmission energy consumption varies with the payload size and the transmitting protocol particularities. It is calculated according to the following equation,

(6) $$E_{tx}=E_{p}\times p_{s},$$

with $E_{tx}$, $E_{p}$ and $p_{s}$ denoting the transmission energy consumption in joule, protocol energy consumption per byte in joule and payload size in bytes, respectively. The payload size $p_{s}$ varies depending on the payload structure, the included services and the message formats. More concretely, it is the summation of the data sizes for all service representations. It is computed as follows,

(7) $$\begin{array}{cc} p_{s}= & \sum_{k=1}^{K}fld\left(s_{k}\right)+\sum_{l=1}^{L}vld\left(s_{l}\right)+\sum_{m=1}^{M}fll\left(s_{m}\right)\\ \; & +\sum_{n=1}^{N}vll\left(s_{n}\right)+\sum_{p=1}^{P}dm\left(s_{p}\right), \end{array}$$

with *s* representing a service and *fld*, *vld*, *fll*, *vll* and *dm* representing the data length in bytes using the fixed-length, variable-length, fixed-length list, variable-length list and data mask format as explained in Section 3.1, respectively. The upper limits of the summations K, L, M, N and P represent the number of services for each format.

## 5. Experimental Evaluation

This section evaluates the proposed IMBC model and its use in a functional scalable heterogeneous IoT system. The system utilised for the experimental evaluation integrates various IoT protocols, namely, LoRaWAN, NB-IoT, WiFi and BLE. The aforementioned protocols support bidirectional communication allowing both server and devices to initiate the communication. The server implements a logically centralised management solution making use of IMBC, which provides interfacing functions for device management. The devices implement the IMBC services and the necessary adaptors to automatically extract and pack meaningful data. To evaluate the proposed communication model, a comparison has been conducted against the LwM2M standard to determine the payload size differences. In addition, we evaluate the energy consumption for two IoT protocols that target data transmission for low power devices.

### 5.1. Services

For the experimental evaluation, five services are transmitted within three different transmission profiles. A summary of the employed services can be found in Table 4. For our experiments, we have implemented three different transmission methods: The first one transmits the raw data, that is, the actual measured data with no additional identifiers for service identification. The second transmission technique utilises the proposed IMBC communication model and transmits the actual measured data together with the service identifiers, which allow extracting and packing meaningful data. The third transmission methods extends upon the second by adding three power profiles that prioritise the five services depending on freely chosen user parameters, such as battery percentage, for example. The power profiles are arbitrarily chosen to exemplify a scenario in which the device configuration is adjusted utilising the IMBC model in order to increase the battery lifetime. More specifically, power profile 1 transmits all service data until the battery capacity reaches 50%. Power profile 2 only transmits data associated with services having priority 0 and 1 until the battery capacity reaches 25%. Last, power profile 3 only sends data having priority 0 until the battery is fully discharged.

The power profiles play an important role for battery-powered devices as it can greatly increase their battery lifetime. Furthermore, and as previously mentioned, the power profiles can be arbitrarily chosen by the user. A key aspect of such an implementation is that one is able to predefine the lifetime of the devices by tuning the profiles. This can greatly aid in planning the battery replacement process, which can be problematic for large scale deployments.

### 5.2. Payload Size

This subsection compares the IMBC and LwM2M Tag-Length-Value (TLV) formats with respect to the generated payload sizes when bootstrapping the services described in Table 4. The values for LwM2M are determined by taking into account that multi-value messages are transmitted following the TLV format. Each service includes the type, the identifier, the length (if applicable) and the value as defined by the LwM2M specification [10]. Note that, in addition to the LwM2M TLV payload, a LwM2M message also comprises the transport bytes imposed by the CoAP protocol; this is not the case for IMBC, which does not rely on CoAP.

Embedding the aforementioned services in a single transmission message generates a message payload size of 36 bytes and 27 bytes for LwM2M TLV and IMBC, respectively. Note that this does not include the transport bytes for LwM2M TLV. The reduction in payload size provided by IMBC, approximately 25%, is significant for battery-powered devices and low-power transmitting protocols since the energy consumption for transmission is proportional to the data to be sent. Moreover, larger payloads may extend over the protocol limitations with regard to the maximum transmission payload size. For example, the maximum payload size for LoRaWAN varies between 51 bytes and 222 bytes for the EU863-870 band channels. In this case, the LwM2M messages may easily pass over the lower limit, therefore making the IMBC model much more suited for LoRaWAN transmitting devices.

Overall, IMBC advances over LwM2M in terms of payload size. Moreover, it eliminates the transport bytes imposed by the CoAP protocol and the need of a LwM2M objects translator for BLE devices as proposed by M. Ha et al. in [16]. In view of the foregoing, we may also conclude that the IMBC model is more suitable than LwM2M for low-power deployments.

### 5.3. Power Consumption

To evaluate the power consumption of IMBC, two IoT protocols have been taken into consideration: LoRaWAN and BLE v5.0. The reason for not incorporating WiFi and NB-IoT in our experiments is justified by the negligible impact of the proposed IMBC identifiers in terms of power consumption per transported bytes, as discussed in [11,18].

#### 5.3.1. LoRaWAN

This subsection evaluates the impact of IMBC in terms of battery lifetime for LoRaWAN enabled devices. The theoretical battery lifetime is computed for a battery capacity of 2400 mAh based on the formalism established in [19]. Specifically, the battery lifetime $T_{lifetime\_unACK}$ for unacknowledged transmission is given by

(8) $$T_{lifetime\_unACK}=\frac{C_{battery}}{I_{avg\_unACK}},$$

with $C_{battery}$ and $I_{avg\_unACK}$ denoting the battery capacity and average current consumption, respectively. $I_{avg\_unACK}$ varies with respect to the notification period and transmission states. It is computed as follows,

(9) $$\begin{array}{c} I_{avg\_unACK}=\frac{1}{T_{Notif}}\sum_{i=1}^{N_{states}}T_{i}\cdot I_{i}, \end{array}$$

with $T_{Notif}$, $N_{states}$, $T_{i}$ and $I_{i}$ denoting the notification period employed by the device, the number of transmission states, the duration of state i and the current consumption of state i, respectively. The average values for the different states are given in [19]. The battery lifetime is computed for three different data rates and the related configurations for EU863-870 band channels. For each data rate, the transmitted payload includes the transmission profiles particularities described below.

- Data rate 0—Spreading Factor 12, Bandwidth 125 kHz,
- Data rate 3—Spreading Factor 9, Bandwidth 125 kHz,
- Data rate 6—Spreading Factor 7, Bandwidth 250 kHz.

**Figure 5:**
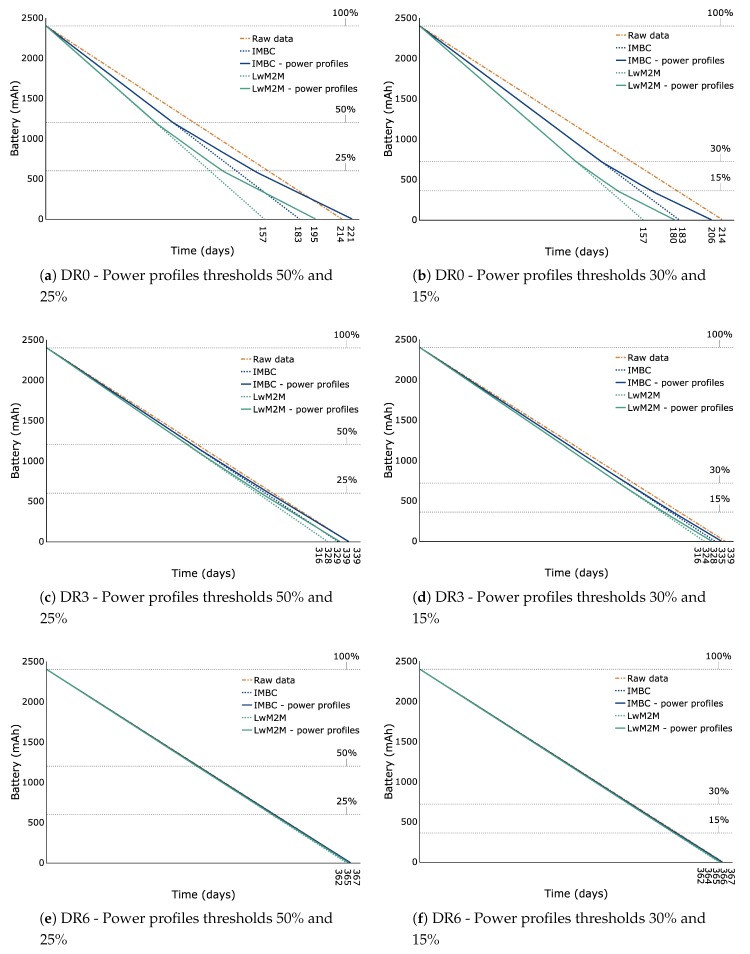
Battery lifetime for LoRaWAN data rates DR0, DR3 and DR6 when transmitting Raw data, IMBC data, IMBC data with power profiles, LwM2M TLV data and LwM2M TLV data with power profiles. The orange coloured lines correspond to Raw data, the blue coloured lines correspond to IMBC data and the green coloured lines correspond to LwM2M data.

Figure 5 demonstrates the relatively limited impact of the IMBC identifiers in terms of power consumption, despite enabling much more functionality with respect to network management. However, note that the impact is more pronounced for lower data rates. Transmitting IMBC data without power profiles utilising Data rate 0 results in a decreased battery lifetime of 14.5% when compared to sending the raw data. For Data rates 3 and 6 the diminished lifetime is reduced to approximately 3.2% and 0.5%, respectively. The negative impact on the battery lifetime, however, can be mitigated by introducing power profiles. When using thresholds 50% and 25% for example, we do not detect any decrease in battery lifetime for IMBC, as shown in Figure 5a,c,e. On the contrary, for Data rate 0 the lifetime increased by more than 3.2% when taking the raw data transmission as the reference.

The same conclusions can be drawn for LwM2M. Also in this case the impact on battery lifetime is more apparent for lower data rates. However, when compared to IMBC, we observe from the figures that the higher payload size of LwM2M negatively impacts the overall power consumption. Though the power profiles indeed mitigate this problem, the thresholds of 50% and 25% do not suffice in this case and the reduced battery lifetime is still substantial, unlike for IMBC.

Figure 5 further shows the results when using power profiles with thresholds 30% and 15%. By comparing the results of using both power profiles thresholds, one can derive the impact of enabling different power profiles. In principle, the results demonstrates the ability to adjust the battery lifetime by tuning the thresholds. Note that this is also true for the highest data rate, where the difference in lifetime is still days. As mentioned previously, the ability to plan and manage the battery lifetime can greatly facilitate and streamline the battery replacement process, which can be very tedious for large scale deployments.

#### 5.3.2. Bluetooth Low Energy v5.0

For BLE v5.0-enabled devices, we compute the theoretical battery lifetime for a battery capacity of 500 mAh by dividing the battery capacity with the estimated average current computed by the power profile estimator published by Nordic Semiconductor [20]. Similarly to LoRaWAN, we incorporate three different data rates in our tests for both IMBC and LwM2M TLV while implementing the same functionalities for both techniques:

- PHY8S—LE Coded S = 8, data rate 128 kbps,
- PHY1—LE 1M, data rate 1 Mbps,
- PHY2—LE 2M, data rate 2 Mbps.

**Figure 6:**
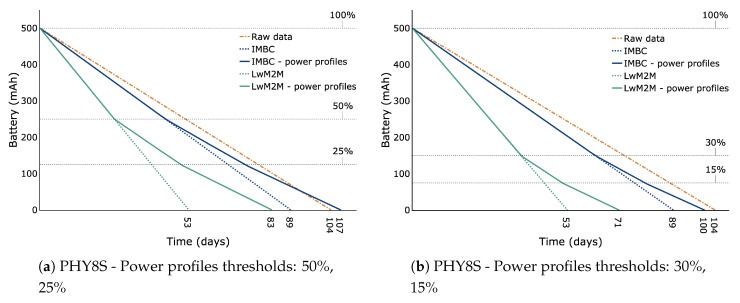

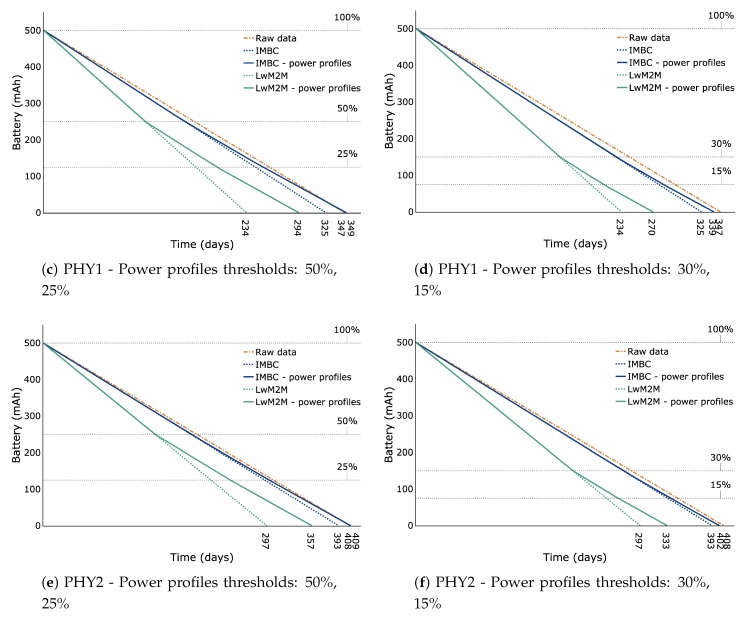
Battery lifetime for Bluetooth data rates PHY8S, PHY1 and PHY2 when transmitting Raw data, IMBC data, IMBC data with power profiles, LwM2M TLV data and LwM2M TLV data with power profiles. The orange coloured lines correspond to Raw data, the blue coloured lines correspond to IMBC data and the green coloured lines correspond to LwM2M data.

The evaluating device is a connected peripheral utilising an nRF52840 chip at a working voltage of 3.3 V with a connection interval of 100 ms and transmission power equal to 0 dBm. Figure 6 illustrates the battery lifetime computed for each data rate and each transmission profile for both IMBC and LwM2M TLV. Generally speaking, the same conclusions as for LoRaWAN can be drawn for the proposed method. Also, in this case, the impact of IMBC without power profiles is greater for lower data rates in terms of battery life. More specifically, for data rates PHY8S, PHY1 and PHY2, the power consumption is increased by approximately 14.5%, 6.5% and 3.7%, respectively, when taking the raw data transmission as a reference. Note that according to [20], values less than 5% are equivalent to device to device variations. For LwM2M TLV without power profiles the increase in power consumption is much more pronounced. In this case, the reduced battery lifetime increased correspondingly by 49%, 33%, and 27%, respectively, when taking the raw data transmission as a reference. Similarly as for LoRaWAN, the increased power consumption can be completely negated by using power profiles with thresholds 50% and 25% when using IMBC, as shown in Figure 6a,c,e. Moreover, they allow us to tune the battery lifetime in a variable window of 18, 24 and 16 days for data rates PHY8S, PHY1 and PHY2, respectively. For LwM2M TLV, however, the reduction in battery lifetime remains substantial even when using power profiles. Taking Figure 6a as an example, we observe a reduction in battery life of 20% for LwM2M TLV. In contrast, IMBC increased battery life time with 2.9%.

## 6. Conclusions

This paper proposed a message-based communication model that achieves network management and functional scalability for heterogeneous IoT systems. The model comprises a dictionary of services utilised by the devices and the server to interact and to perform the desired actions agnostic to the implied IoT protocol. Syntactic and semantic interoperability is achieved by implementing adaptors that automatically interpret data meaningfully and convert the service messages into system-readable representations. This paper described the message formats, the management procedures and the interfacing functions introduced by the proposed communication model, and addressed the model’s particularities in terms of network management and functional scalability. We evaluated the proposed methods and showed its applicability in a functional scalable heterogeneous IoT system that integrates LoRaWAN, NB-IoT, WiFi and BLE. Our experimental assessment revealed that our method allows for more compact data representation than that of the LwM2M standard, a protocol specifically designed with IoT applications in mind. Our experiments also revealed that our communication model has a limited impact in terms of power consumption, despite providing much more functionality with respect to network management. In fact, the increased power consumption can be completely negated when using the power profiles implemented on top of our communication model. Besides reducing the energy consumption, those power profiles allow one to predefine the battery lifetime of the devices, which can greatly aid in streamlining the battery replacement process in large scale deployments. Regarding future work, the underlying transmitting protocols impose limitations with respect to the payload size. In the current approach, the employed transmission protocol is predefined. Using the appropriate transmission protocol depending on the payload size is left as a topic for future investigation.

## Figures and Tables

**Table 1:** Similarities and differences between IMBC and LwM2M.

	IMBC	LwM2M
Service bootstrapping	Yes	Yes
Payload size	Service dependant *	Higher for most services *
Network management	Yes	Yes
Device identification	No	Yes
IP required	No	Yes

* As described in Section 5.2.

**Table 2:** Semantic data services categories.

Identifier Range	Service Type	Description
00-0F	Message Data Object (MDO)	Message specific services (e.g., device configuration, transmission cycle, error messages, timestamp)
10-1F	Network Management (NMT)	Transmission protocol transition, firmware update, device control (power off/reset/sleep)
20-BF	Payload Data Object (PDO)	Payload data (e.g., temperature data, humidity data, etc.), custom messages
C0-FF	Reserved (RSV)	Reserved for future implementations

**Table 3:** Device action options, Protocol options and error messages. The considered protocols include Low Range Wide Area Network (LoRaWAN), Narrowband IoT (NB-IoT), WiFi and Bluetooth Low Energy (BLE).

Action	Identifier	Protocol	Identifier	Error message	Identifier
Power off	00	Auto	00	Message parsing error	00
Reboot	01	LoRaWAN	01	Protocol not implemented	01
Sleep	02	NB-IoT	02	Protocol connection failed	02
		WiFi	03	Firmware update error	03
		BLE	04	Service not implemented	04
				Transmission cycle setup error	05

**Table 4:** Service data priority and the payload size of the raw data, the IMBC data and the LwM2M TLV data.

Service Data	Priority	Raw Data Size	IMBC Data Size	LwM2M TLV Data Size
Battery level	0	1 bytes	2 bytes	3 bytes
Temperature #0	0	4 bytes	5 bytes	6 bytes
Temperature #1	1	4 bytes	5 bytes	6 bytes
Humidity	1	1 bytes	2 bytes	3 bytes
Custom message	2	9 bytes	12 bytes	12 bytes
Total bytes		19 bytes	26 + 1 * bytes	30 + 6 * bytes

* Note that additional bytes are required to send multiple service values (e.g., Temperature #0 and #1).
